# Prenatal Exome Diagnostic Yield, Syndromic Landscape and Secondary Findings

**DOI:** 10.1002/mgg3.70207

**Published:** 2026-04-07

**Authors:** Kayleigh Avello, Shawn Gessay, Megan Nelson, Connie Schultz, Natalie Jacob

**Affiliations:** ^1^ Prevention Genetics LLC, a Wholly Owned Subsidiary of Exact Sciences Corporation Marshfield Wisconsin USA; ^2^ University Health Texas USA

## Abstract

**Objective:**

Exome sequencing (ES) has become increasingly more prevalent across maternal fetal medicine spaces when anomalies are visualized on ultrasound. As such, Prevention Genetics implemented secondary finding categories into their prenatal exome based on professional recommendations in June of 2021. This study aimed to determine the diagnostic yield and syndromic landscape of reported variants during that timeframe.

**Methods:**

Prenatal ES cases from June 2021 to June 2023 were retrospectively reviewed for secondary finding opt‐ins, overall result outcomes, variant nomenclature, and inheritance/segregation patterns. From this data, quantitative and descriptive statistical methods were used to determine the frequency of positive primary and secondary results, including cases in which multiple molecular diagnoses were detected.

**Results:**

Of 520 fetal samples included in the cohort, 131 (25.2%) had a positive molecular result consistent with the clinical indication for exome. Within those 131 cases, 81 (61.8%) were caused by a *de novo* variant. From the total 520‐sample cohort, 260 (50%) cases opted into at least one SF category. Among the 222 fetuses tested in which ACMG guideline‐recommended genes were opted into, 14 (6.3%) were found to carry a reportable variant in genes associated with cardiovascular, metabolic, pediatric tumor‐predisposition, or connective‐tissue disorders. Four fetuses in the COD opt‐in cohort were discovered to carry a deleterious variant in two separate SF genes, constituting comorbid pediatric molecular diagnoses. Unlike the primary reporting category, the majority (*N* = 25/30, 83.3%) of COD SF variants were inherited from a carrier parent.

**Conclusion:**

The aim of this study is to provide meaningful information that can aid in the exome consenting process as improvements in testing technologies and laboratory offerings evolve in response to developments in genetic understanding and professional guidelines. As such, it is crucial for healthcare teams to stay abreast of the increased scope of testing, potential test outcomes, and the phenotypic expansion of various genetic disorders.

## Graphical abstract

The aim of this study is to provide meaningful data surrounding prenatal diagnostic and secondary finding results that can aid in the exome consenting process. As an emerging area of genomic medicine, these results provide holistic incidence rates for rare genetic conditions, presently understudied in the prenatal realm.

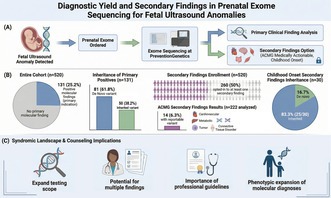

## 1 Introduction

It is estimated that approximately 2%–4% of pregnancies present with major fetal anomalies, which can be isolated or found in conjunction with a constellation of other features that range in clinical severity (Persson et al. 2017). The characterization of anomalies and providing the option of appropriate diagnostic investigations is a mainstay of prenatal genetics, with the purpose of understanding the underlying etiology in addition to guiding pregnancy and/or neonatal management (Cargill and Morin 2017). Exome sequencing (ES) has become increasingly more prevalent across a variety of clinical settings, including maternal fetal medicine (MFM) practices, when fetal anomalies are visualized on ultrasound. ES at Prevention Genetics assesses the coding regions of nearly all clinically relevant genes, in addition to the adjacent intronic regions. Though a valuable diagnostic tool for various fetal indications, challenges complicate widespread implementation of ES. These challenges include the ability to sequence and analyze full ES datasets timely enough to allow for downstream pregnancy management, as well as the ability to reconcile variants with reduced penetrance, variable expressivity, and/or those that are of uncertain clinical significance. Further, while there is a large effort to understand the prenatal presentation of many Mendelian disorders, not all disorders present in the fetal period (Wapner et al. 2012), nor to date are all recognizable fetal anomalies documented in the literature with an established molecular etiology.

In an effort to overcome the challenges associated with prenatal ES, commercial laboratories prioritize reporting variants most likely associated with the fetal phenotype. At PreventionGenetics, ES is only performed in the setting of a fetal anomaly. The inclusion of detailed clinical information is crucial for the ability to select variants that may contribute to the molecular etiology creating the phenotype. While the main focus and use of ES in the prenatal setting has been to aid in prenatal diagnosis, with improvements in NGS and bioinformatics pipelines, as well as increased knowledge of gene‐disease associations, the American College of Medical Genetics and Genomics (ACMG) expanded its recommendations for prenatal ES reporting to include secondary finding (SF) opt‐in reporting categories. (Monaghan et al. 2020) In observance of the ACMG's guidelines, PreventionGenetics offers two secondary finding categories with its prenatal ES products: (1) ACMG guideline recommended gene list and (2) genes associated with childhood onset disorders (COD). The ACMG guideline recommended gene list includes those associated with medically actionable findings, primarily cardiovascular and hereditary cancer genes (Miller et al. 2022). While there is not a defined list for childhood onset disorders from ACMG, this reporting category aims to assess for highly penetrant genes that cause moderate to severe disorders presenting in the pediatric period (Monaghan et al. 2020). Both secondary finding categories will only report out pathogenic or likely pathogenic variants that fit the Mendelian pattern for that gene/disorder. Due to the nature of ES, there is potential for the identification of variants with implications that are unrelated to the primary indication for testing (Austin‐Tse et al. 2022); therefore, it is important to note that secondary findings are part of a predefined reporting category that patients can opt into at the time of ordering and consenting, while incidental findings are not related to the proband phenotype nor are part of the predefined selected secondary finding category.

Given the recent addition to the test analysis offerings in the last few years, as well as a shift in professional recommendations, this study aims to understand the overall diagnostic yield for prenatal ES cases alongside the uptake and incidence of secondary findings for applicable orders. Additionally, this dataset seeks to draw attention to important questions about pre‐ and post‐test considerations for patients and clinicians alike.

## 2 Methods

Data at PreventionGenetics from prenatal ES cases performed from June 2021 to June 2023 was retrospectively reviewed to determine overall diagnostic yield in addition to the syndromic landscape of both phenotypically relevant and secondary findings. Cases were assessed to determine if a fetal analysis had either the ACMG or COD SF option selected, if a variant was reported in either category, and if a definitive causative variant(s) was found for the clinical indication of testing based on the presenting fetal phenotype shared with the lab at the time of testing.

## 3 Results

### 3.1 Demographics

In this cohort (*N* = 520), maternal age ranged from 18 to 48 years, with a median age of 33 years. The largest proportion of pregnant women were between the ages of 31–36. Based on the credentials provided on the requisition form in this cohort, a genetic counselor (GC) was involved in placing 93.3% of prenatal ES orders; the remaining cases were facilitated by a physician only. The majority of tests were ordered as a trio analysis (80.8%), followed by duo requests (10%), then singleton cases (9.2%).

### 3.2 Positive Primary Finding Cases

In this cohort of 520 fetuses, 131 (25.2%) had a primary finding result and only one fetus was discovered to have dual molecular diagnoses. Overall, the PreventionGenetics prenatal ES molecular diagnostic rate varies by the inclusion of comparators, generally biological parents, to assist in the analysis of proband data. The diagnostic rates for each family testing structure are as follows: Trio—26%, Duo—14%, Singleton—25%. With respect to the analysis, the vast majority of fetal cases had more than 1 HPO term provided (*N* = 120) compared to 11 cases where only a single HPO term was noted for test indication. Single HPO term cases included Dandy‐Walker malformation, increased nuchal translucency, hydrops fetalis, micrognathia, microcephaly, situs inversus totalis, hypoplastic left heart, and abnormal genitalia. In this cohort, the clinical category represented by the respective fetal phenotype included multiple malformations (*N* = 53) as the most common indication for testing, followed by skeletal (*N* = 29) and neurological (*N* = 16) ultrasound findings.

**FIGURE 1:**
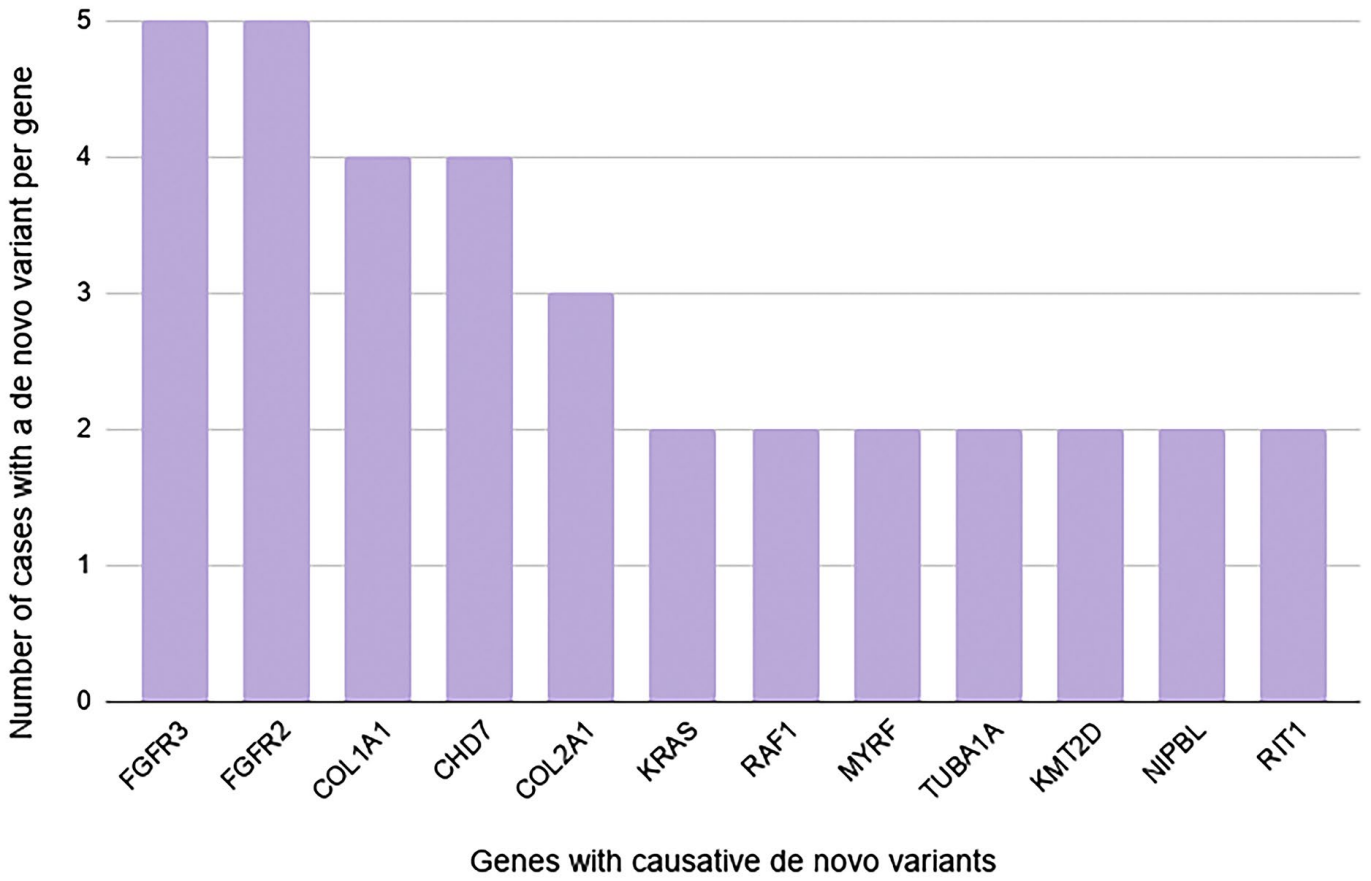
Primary findings positive results—top *De Novo* genes.

Most positive cases were due to a *de novo* variant in an autosomal dominant gene (*N* = 81, 61.8%) (Figure 1), compared to recessive disorders (*N* = 22, 16.8%) or disorders (AD/XL) known to be inherited from a single parent (*N* = 10, 7.6%) (Table 1). For the remaining cases, the applicable inheritance could not be determined based on the testing structure.

**TABLE 1:** Volume of fetal primary finding cases by variant parent of origin and condition mode of inheritance.

Parent of Origin (MOI)	Number of cases
*De Novo* (AD)	81
Both Parents Heterozygous (AR)	19
Unknown (AD)	11
Not Maternal (AD)	5
Maternal (X‐linked)	6
Paternal (AD)	3
One Maternal/One Not Maternal (AR)	1
Unknown (X‐linked)	1
Not Paternal (AD)	1
*De Novo*/Maternal (AR)	1
Paternal (X‐linked)	1
Unknown (AR)	1
Total	131

### 3.3 Secondary Findings

Providers requested reporting of secondary findings in 50% (*N* = 260/520) of orders; both SF categories had an increase in uptake over the 2‐year period. Of those that opted in for secondary findings, most providers requested COD and ACMG SF categories be reported (69.2%, *N* = 180/260) compared to just one SF category. In this cohort, 16.2% (*N* = 42/260) of orders opted into just the ACMG guideline recommended gene list while 14.6% (*N* = 38/260) of orders opted into just the childhood onset disorders category. The majority of SF variants were inherited from a parent (*N* = 37) and a small number of variants were found to be *de novo* (*N* = 4). Of interest, there was one fetus identified to have a finding in both secondary finding categories.

### 3.4 Positive Secondary Finding Cases

#### 3.4.1 ACMG

**FIGURE 2:**
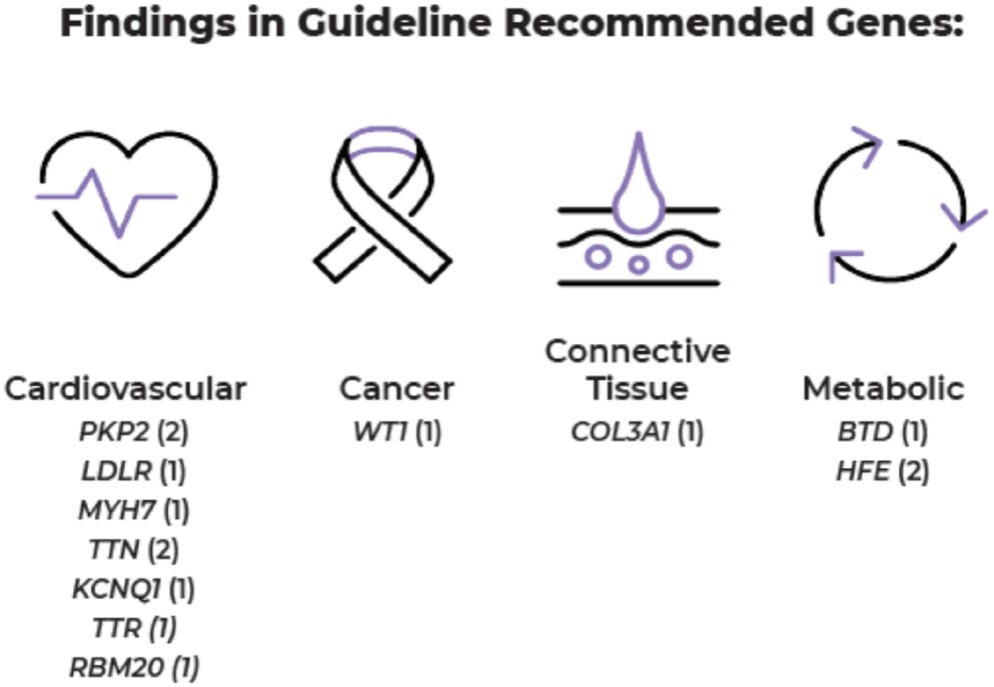
Findings in ACMG guideline genes.

Of the 222 fetuses tested whose analysis included the guideline recommended genes, 14 (6.3%) were found to carry a likely pathogenic or pathogenic variant in genes associated with either a cardiovascular, metabolic, pediatric tumor predisposition, or connective tissue disorder (Figure 2). Of note, there were no fetuses found to have a causative variant associated with an adult‐onset hereditary cancer syndrome. There were 12 probands that inherited the variant of interest from a parent, one fetus had a variant arise *de novo*, and one fetus had a variant of unknown inheritance as parents were not included in the analysis of proband data.

#### 3.4.2 COD

**FIGURE 3:**
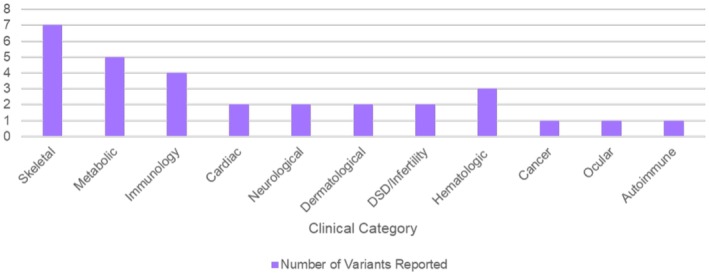
COD secondary finding variants by clinical category.

Of the 218 fetuses tested whose analysis included the childhood onset genes, 26 (11.9%) were found to carry a likely pathogenic or pathogenic variant. Four fetuses carried two different gene variants in the COD SF category. With regards to inheritance, the majority of the COD SF variants were passed on from a parent (*N* = 25/30) (Table 2). The top three clinical categories reported as part of this secondary finding reporting option were skeletal, metabolic, and immunologic, respectively (Figure 3). Many of the conditions identified by the COD SF category are those that would not have been captured with prenatal screening, such as ultrasound.

**TABLE 2:** Volume of COD variants by parent of origin and condition mode of inheritance.

Parent of origin (MOI)	Number of variants reported
Maternal (AD)	12
Paternal (AD)	8
*De Novo* (AD)	3
Both Parents (AR)	2
Maternal (X‐linked)	3
Unknown Inheritance (AD)	2
Total N^a^	30

^a^ 4 fetuses carried two different variants in the COD SF category.

## 4 Discussion

### 4.1 Positive Primary Finding Cases

Overall, this cohort was found to have a diagnostic rate of approximately 25%, which is consistent with the yield quoted in current literature (Slavotinek et al. 2023). Notably, the diagnostic yield was highest when the analysis was performed as a trio, most often with the parental samples as comparators. This demonstrates the utility in leveraging parental data to readily identify *de novo* variants of interest as well as to ascertain the phase of homozygous and compound heterozygous variants for autosomal recessive conditions. While the diagnostic yield of a singleton analysis was comparable to a trio in this cohort, the diagnostic rate for the duo remained lowest. Presumably, this may be due to a low N in this dataset that was performed as duos, but further studies would be needed to clarify the significance of this finding.

While multiple malformations were the most common indication for prenatal ES in this cohort, there were several fetuses that had only a single ultrasound finding at the time of sample collection. This highlights that apparently isolated anomalies can benefit from ES since a large proportion of monogenic conditions can present with variable expressivity across the lifespan, including in the prenatal period. Further, one phenotype can be the result of a number of genetic etiologies that may not be fully represented by one predefined panel, such as increased nuchal translucency. As a result, ES allows for a thorough and comprehensive analysis of most known genetic etiologies while being mindful of turnaround time. Performing panels reflexively and in a stepwise fashion may not allow for the expedited return of information and could lead to a missed diagnosis as the result of panel curation bias.

Given most modes of inheritance were autosomal dominant and arose *de novo*, this data demonstrates the importance of informed and comprehensive patient education and counseling regarding prenatal screening offerings and residual risks. In this dataset, 9 of the top 12 genes with *de novo* variants are included on single gene NIPT currently: *FGFR3, FGFR2, CHD7, COL1A1, KRAS, RAF1, RIT1* and *NIPBL*. This highlights the utility of the single gene non‐invasive prenatal screening (NIPS) when patients decline invasive, diagnostic testing in the setting of fetal ultrasound anomalies (Guseh et al. 2021). While the screen would not confer a molecular diagnosis and confirmatory testing should be pursued, the screen can help guide care and targeted follow‐up testing if the screening result is consistent with the fetal structural findings. Of the overall 131 positive cases, 81 (61.8%) were due to a *de novo* variant, indicating a minimal reproductive risk for the parents, but germline mosaicism cannot be ruled out. In those respective cases, carrier screening of recessive disorders would have been uninformative for the positive finding on exome; this also applies to the rare single case where an autosomal recessive disorder had a *de novo* variant arise on the opposite allele when only one parent was identified as a carrier.

Prenatal ES, particularly with the inclusion of secondary findings, will allow for reverse phenotyping. This can lend way for a more robust knowledge base surrounding the fetal presentation of Mendelian disorders, thereby allowing for more syndromes to be readily recognized via prenatal ultrasound and increasing the diagnostic rate of molecular testing over time. In addition, prospective identification of disorders will allow for a better understanding of the clinical features as they present across ages.

### 4.2 Positive Secondary Finding Cases

Half of the orders for prenatal ES in this cohort opted into at least one secondary findings category, and the vast majority of these orders were known to have genetic counselor involvement. Further, the uptake of secondary findings grew over the course of the two‐year study. This seems to illustrate quick adoption of professional guidelines put out by ACMG as well as clinicians' willingness to introduce the topic of secondary findings into pre‐test consenting conversations. Of note, a genetic counselor was involved in the ordering of all cases that had a positive secondary finding result.

In this cohort, the majority of SFs from both categories were inherited from a parent, though it is unknown whether families were aware of their molecular status. Between the two SF categories, reportable variants were seen across clinical categories where a fetal indication generally would not be expected; this can highlight the benefit of ascertaining this information prior to symptom emergence to help guide neonatal and pediatric care. In cases where parental inheritance could be assessed, the majority of SF variants were inherited, though the clinical status of most parents was unknown.

When examining the positive secondary finding cases specifically, 18 fetuses had a negative result respective to the indication for testing, highlighting that a molecular cause for the presenting fetal phenotype was not ascertained. Potential explanations for a negative primary finding may include: (1) the fetus has a genetic condition without an established molecular cause (2) the molecular finding falls outside the limits of detection with current technology (3) the fetus is presenting with a multifactorial condition or (4) as stated above, the secondary finding result may be reflective of the primary cause of disease but the presenting fetal phenotype has not yet been associated with that particular disorder. From this cohort, a large percentage of patients opted in for the COD category, which potentially was viewed as a way to catch clinically relevant variants not completely associated with the fetal phenotype, since not all prenatal findings have been clearly described for rare pediatric genetic conditions.

This data highlights that there may be a larger percentage of pregnancies affected with a genetic condition, as well as a wider phenotypic spectrum for many monogenic disorders not clearly known to date based on what has been documented in the literature (Best et al. 2018). With the addition of SFs being offered prenatally, it does bring up challenges with respect to data analysis. Certain genes have complex disease associations and multiple modes of inheritance, with clinical features that can present across age ranges. With the ACMG medically actionable gene list being dynamic and updated regularly, commercial laboratories must update their filtering pipelines to account for these changes. There is potential for the diagnostic yield to change over time as NGS coverage improves, and more is understood regarding variant curation and current variants of uncertain significance. The definition of childhood onset has a broad and subjective meaning as well, making it difficult to create a specified gene or condition list, which in turn creates challenges for the care team in counseling and consenting patients regarding the potential information that would be uncovered. Further, as the ACMG guideline recommended gene list contains both childhood and adult‐onset conditions, counseling for this secondary finding category must account for that aspect of identification of adult‐onset conditions in both a fetal proband as well as a potential presymptomatic parent. In addition, there are considerations that should be taken into account when facing the implementation of secondary findings into the larger healthcare system. For instance, the addition of more reporting categories and genes leads to increased lab resources and applicable time spent on a single case, which has the potential to increase turnaround time as well as test cost. When scaling the implementation of these recommendations, thought and consideration is needed surrounding lab resources such as additional variant confirmations and interpretation time; more reporting categories and secondary finding options makes the genetic counseling and patient management aspects more challenging, especially if the conditions are not expected to present for several years following birth.

Overall, prenatal ES has the ability to provide more information across genetic etiologies, highlight the differences in phenotypic expression from a fetus to parents, and provide the opportunity for cascade testing of at‐risk family members when genetic diagnoses are made. Further, there are benefits in both the reproductive and pediatric genetic spaces when implementing secondary finding options into prenatal ES. In the reproductive genetics space, prenatal ES can have an impact on pregnancy management decisions and delivery of care. Results from prenatal ES can also assist with future reproductive planning for parents by providing accurate recurrence risk information or targetable loci for both prenatal diagnostics and preimplantation genetic testing. In the pediatric space, prenatal ES with secondary findings can provide a better prognosis and treatment options that may include clinical trial enrollment, dietary changes, surgeries, and/or management by specialized care teams, even before symptoms manifest. Further, the identification of presymptomatic diagnoses is in line with a precision medicine approach and could overall reduce the extraneous health care costs associated with identifying a unifying diagnosis early on in life.

This dataset provides evidence to aid in genetic counseling, informed consent, and patient empowerment as well as offers insight into clinical care, including the transition from prenatal to pediatric care.

## 5 Limitations and Future Directions

Although there have been many advancements with prenatal screening and diagnostic testing, fetal HPO terms are still currently limited since genetic conditions have not previously had well‐described fetal findings in the literature. As a result, the analyses performed are only as robust as the prenatal evidence available surrounding clinical presentations seen for applicable disorders. Additionally, fetal secondary findings are still an emerging reporting category available to families. Given this, further research over time will be needed to determine how this data compares to postnatal secondary finding positive rates, as well as its impact on medical management across the life span.

## Author Contributions

All listed authors have contributed to the manuscript substantially and have agreed to the final submitted version. Each author helped with data collection, analysis, presentation ideas, and manuscript preparation.

## Funding

The authors have nothing to report.

## Ethics Statement

The authors have nothing to report.

## Consent

The authors have nothing to report.

## Conflicts of Interest

At the time of this study, Kayleigh Avello, Shawn Gessay, Natalie Jacob, Connie Schultz, and Megan Nelson were employees of PreventionGenetics LLC, a wholly owned subsidiary of Exact Sciences Corporation. These authors owned stocks with Exact Sciences.

## Data Availability

Data sharing not applicable to this article as no datasets were generated or analyzed during the current study.
